# Supplementary material for: ADAMTS4 is a crucial proteolytic enzyme for versican cleavage in the amnion at parturition
Source: Commun Biol. 2024 Mar 9;7:301. doi: 10.1038/s42003-024-06007-w (PMC10924920; doi:10.1038/s42003-024-06007-w)
Supplement: Supplementary file 2 — Supplementary information [file 42003_2024_6007_MOESM2_ESM.pdf]

Supplementary information for

ADAMTS4 is a crucial proteolytic enzyme for versican cleavage in the amnion at parturition

Meng-Die Li[1,2], Jiang-Wen Lu[1,2], Fan Zhang[1,2], Wen-Jia Lei[1,2], Fan Pan[1,2], Yi-Kai Lin[1,2], Li-Jun Ling[3], Leslie Myatt[4], Wang-Sheng Wang[1,2*], Kang Sun[1,2*]

*To whom all correspondence should be addressed:

Dr. Kang Sun, sungangrenji@sjtu.edu.cn or Dr. Wang-sheng Wang, wangsheng_wang@hotmail.com, Center for Reproductive Medicine, Ren Ji Hospital, School of Medicine, Shanghai Jiao Tong University, Shanghai, P.R. China. Tel: (86)2120284551

Contents:

Supplementary Fig. S1 Comparison of the hyalectan family member expression in the human amnion

Supplementary Fig. S2 Changes of *ADAMTS4* and *LRP1* transcripts in the human amnion in deliveries with SROM as measured with transcriptomic sequencing.

Supplementary Fig. S3 Comparison of LRP1 protein abundance in cultured amnion epithelial and fibroblast cells as measured with Western blotting.

Supplementary Fig. S4 Effect of inflammatory factors on *LRP1* mRNA in human amnion fibroblasts.

Supplementary Fig. S5 Effect of IL-1β on VCAN degradation in the presence and absence of the ADAMTS4 inhibitor.

Supplementary Fig. S6 The knockdown efficiency of LRP1 in human amnion fibroblasts.

Supplementary Fig. S7-24 Uncropped and unedited gel images

Supplementary Table S1 Primer sequences used in qRT-PCR

a

AccID	Sample1	Sample2	Sample3	Sample4	Sample5	Sample6
VCAN	31.50	12.43	22.79	4.49	6.29	18.55
ACAN	0.16	0.02	0.18	0.01	0.16	0.13
NCAN	0	0	0	0.02	0	0.02
BCAN	0	0.01	0	0	0.02	0

b

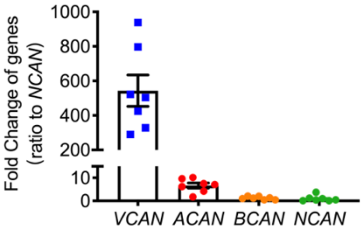

Supplementary Fig. S1 Comparison of the hyalectan family member expression in the human amnion. a Comparison among *VCAN, ACAN, NCAN* and *BCAN* gene transcripts (FPKM, Fragments Per Kilobase of transcript per Million mapped reads) in the human amnion collected from term pregnancies irrespective of labor status (n=6) as measured with transcriptomic sequencing. **b** qRT-PCR analysis showing that *VCAN, ACAN, NCAN* and *BCAN* mRNA abundance in the human amnion collected from TNL (n=7). Data are mean ± SEM.

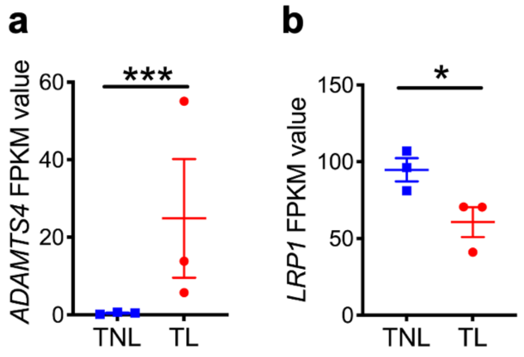

Supplementary Fig. S2 Changes of *ADAMTS4* (a) and *LRP1* (b) transcripts in the human amnion in deliveries with SROM as measured with transcriptomic sequencing. TNL, term non-labor; TL, term labor. Data are mean ± SEM. Statistical analysis was performed with Mann–Whitney U test. *$p<0.05$, ***$p<0.001$ vs. TNL. n=3.

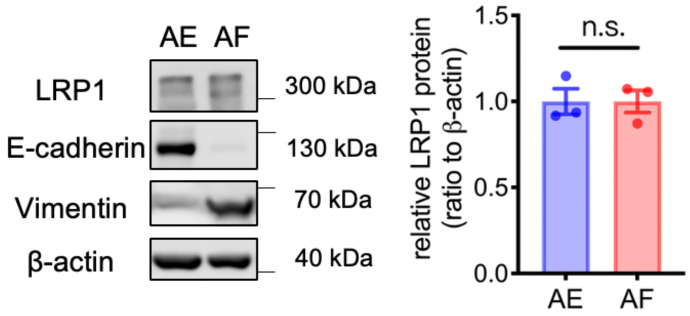

Supplementary Fig. S3 Comparison of LRP1 protein abundance in cultured amnion epithelial and fibroblast cells as measured with Western blotting. AE, amnion epithelial cells; AF, amnion fibroblasts. E-cadherin and Vimentin are markers of amnion epithelial cells and fibroblasts respectively. Data are mean ± SEM. Statistical analysis was performed with paired Student's t-test. n.s., no significance. n=3.

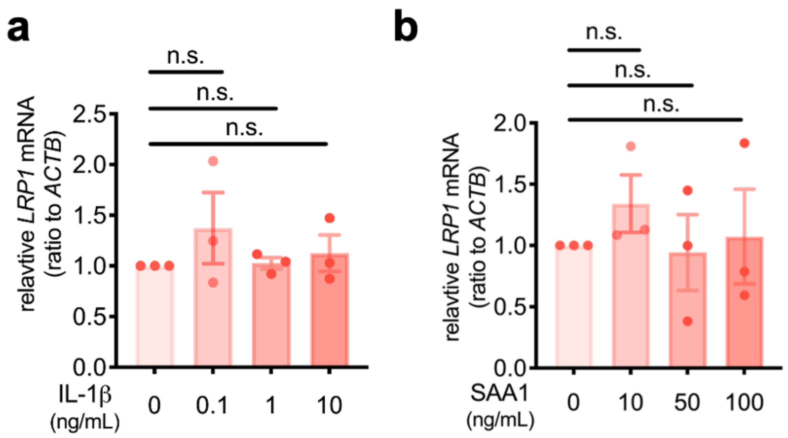

Supplementary Fig. S4 Effect of inflammatory factors (a and **b) on *LRP1* mRNA in human amnion fibroblasts.** Data are mean ± SEM. Statistical analysis was performed with one-way ANOVA test followed by Newman-Keuls multiple-comparisons test. n.s., no significance. n=3.

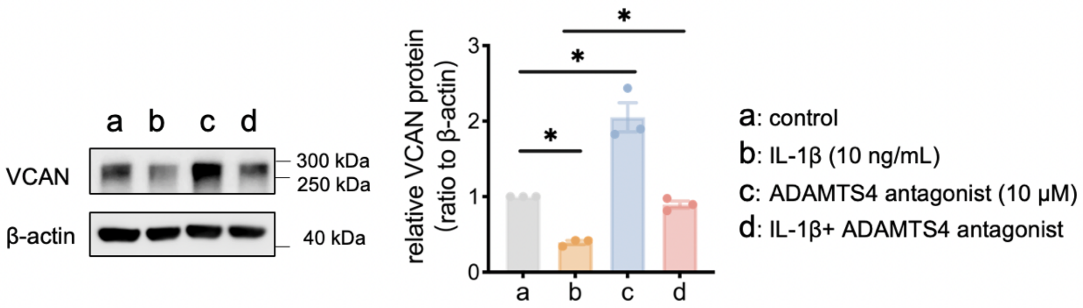

Supplementary Fig. S5 Effect of IL-1β on VCAN degradation in the presence and absence of the ADAMTS4 antagonist. The induction of degradation of VCAN by IL-1β (10 ng/mL, 24 hours) was partially blocked by the ADAMTS4 antagonist (10 μM, 24 hours). Data are mean ± SEM. Statistical analysis was performed with one-way ANOVA test followed by Newman-Keuls multiple-comparisons test. *$p<0.05$. n=3.

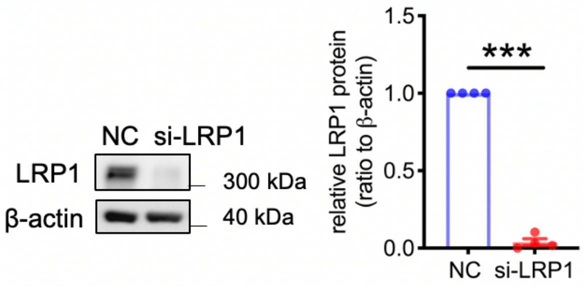

Supplementary Fig. S6 The knockdown efficiency of LRP1 in human amnion fibroblasts. The knockdown efficiency of LPR1 is about 95%. Data are mean ± SEM. Statistical analysis was performed with paired Student's t-test. ***$p<0.001$ vs. NC (negative control). n=4.

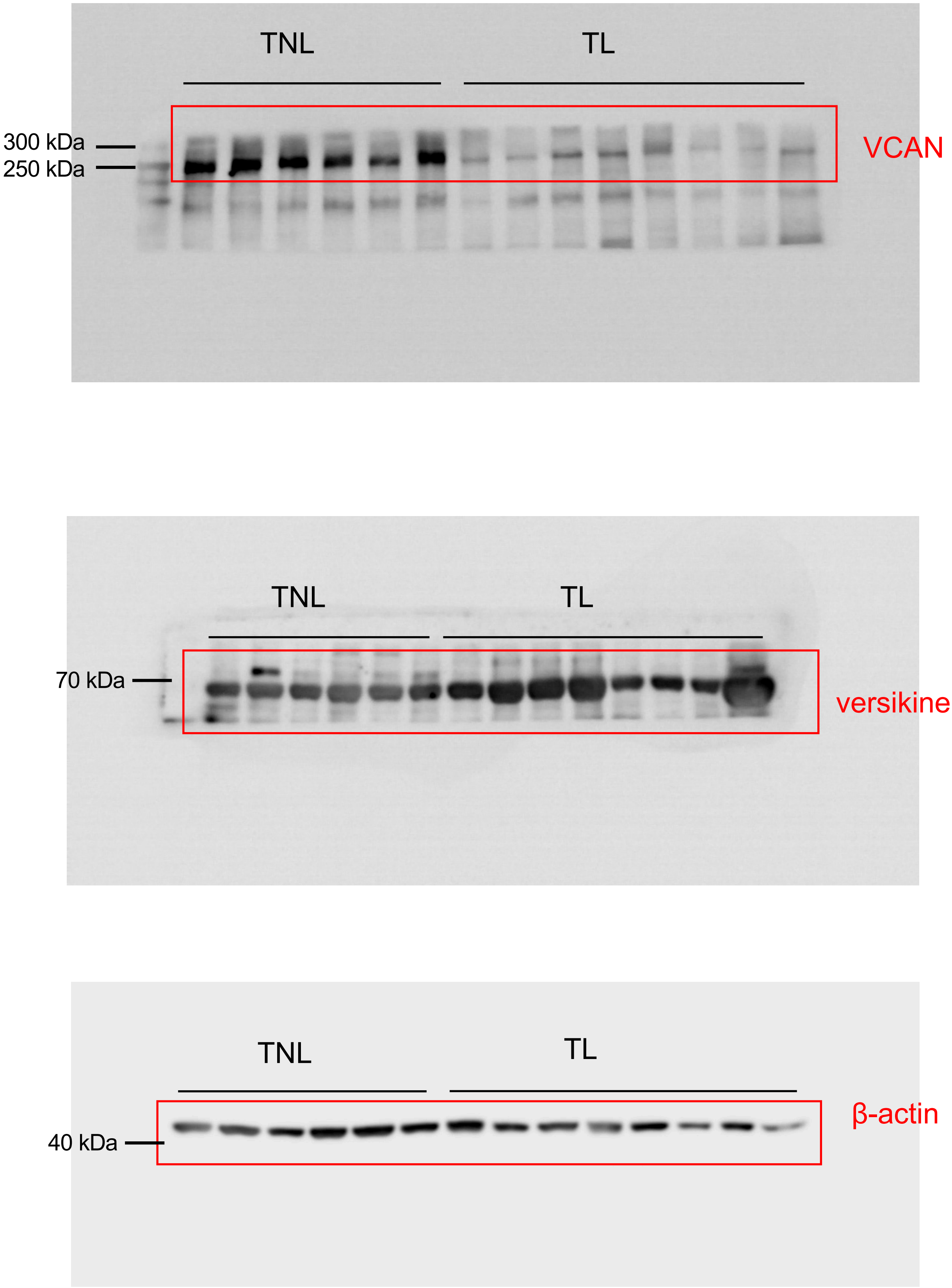

Supplementary Fig. S7: Uncropped and unedited gel image for Fig. 1c.

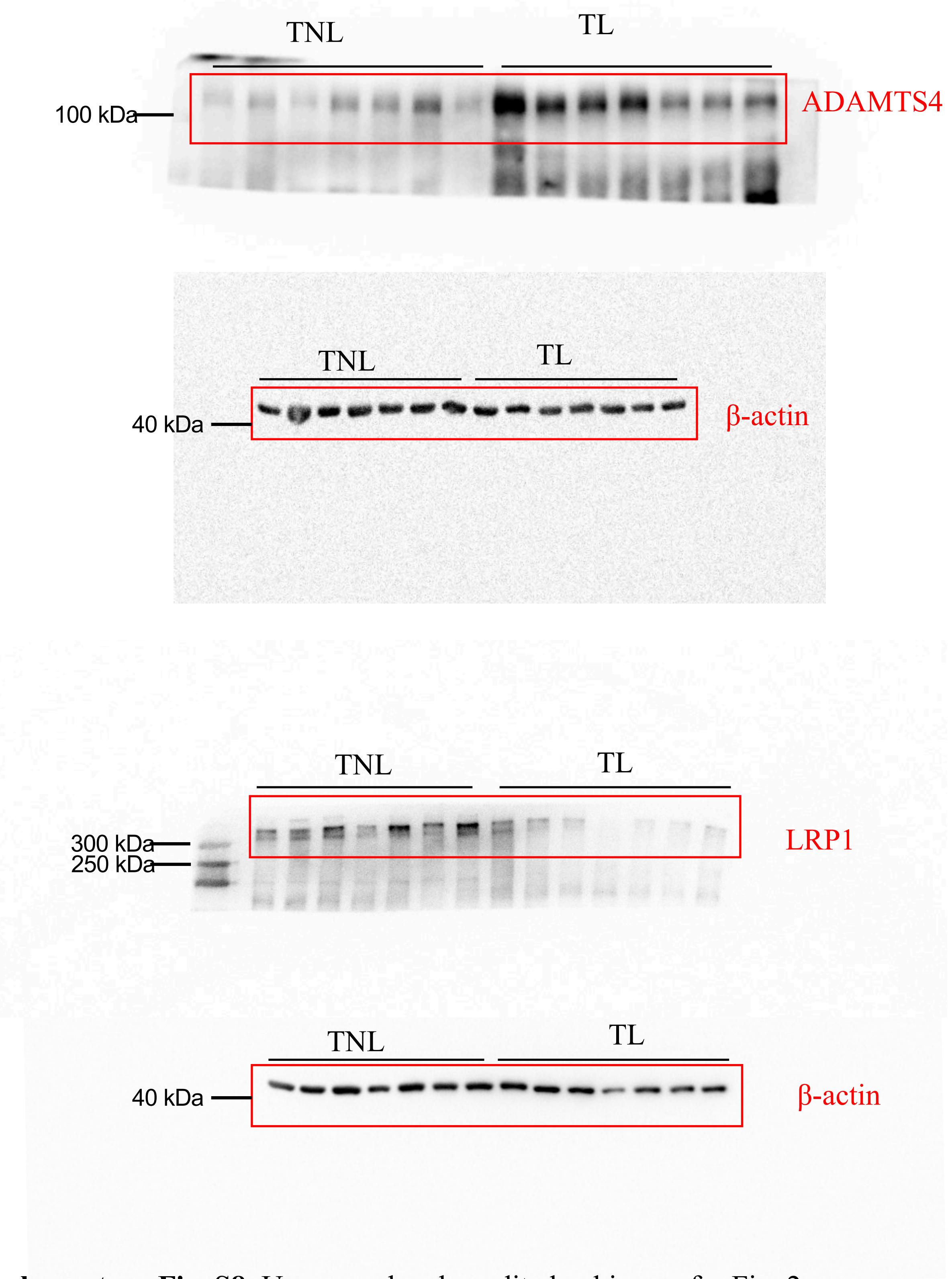

Supplementary Fig. S8: Uncropped and unedited gel image for Fig. 2c.

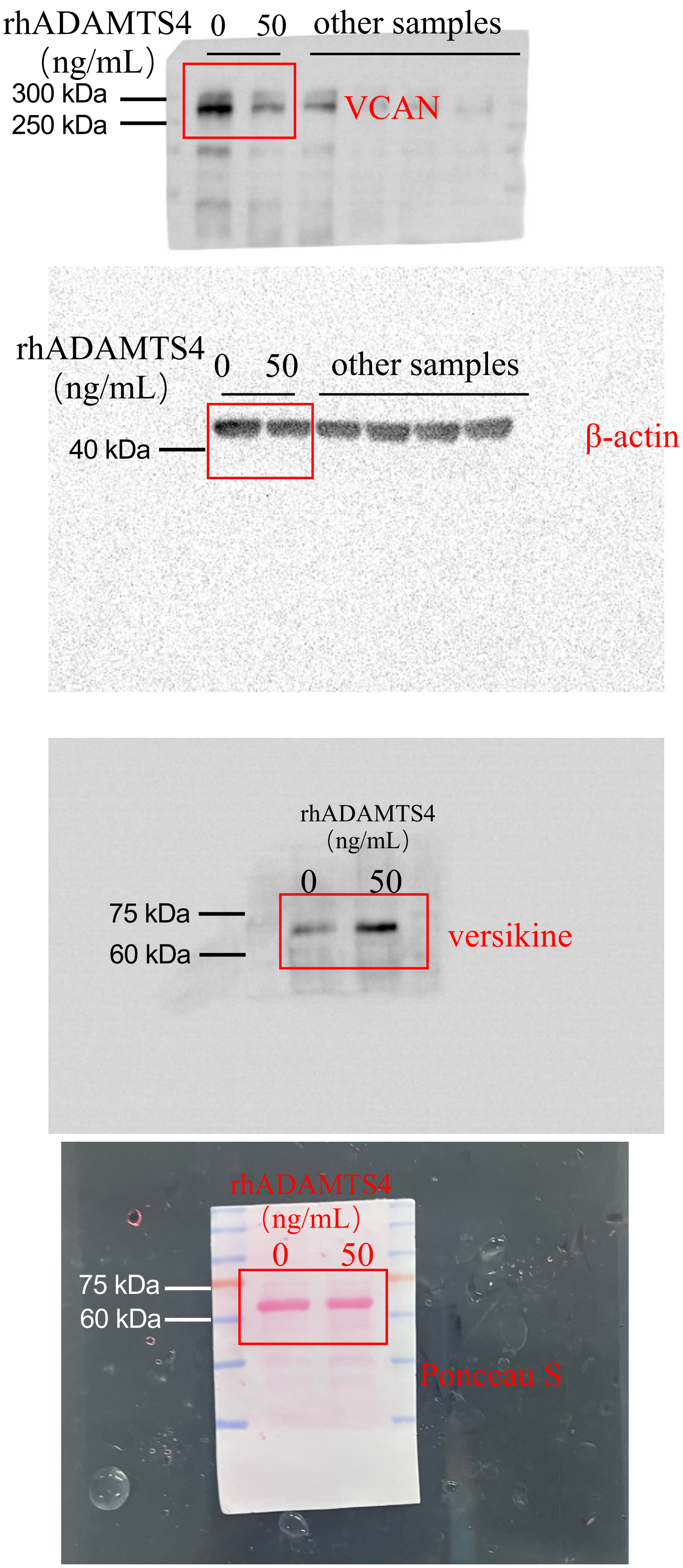

Supplementary Fig. S9: Uncropped and unedited gel image for Fig. 3b.

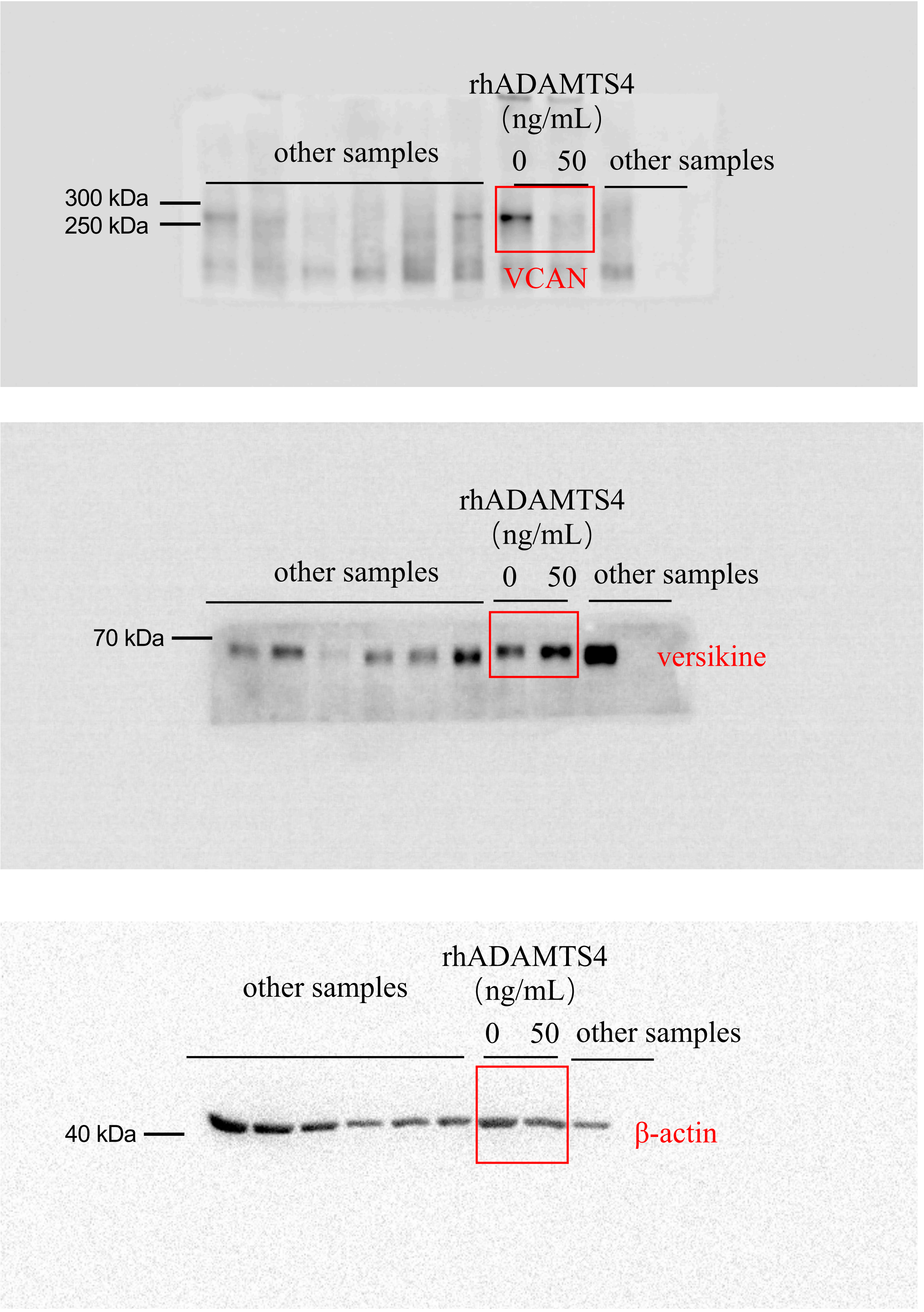

Supplementary Fig. S10: Uncropped and unedited gel image for Fig. 3d.

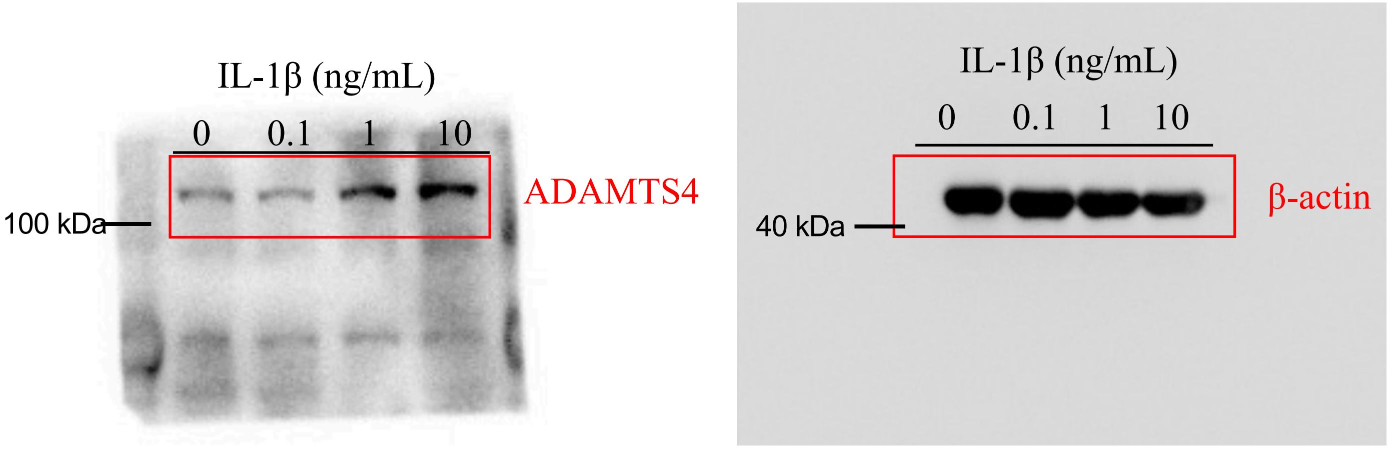

Supplementary Fig. S11: Uncropped and unedited gel image for Fig. 4b.

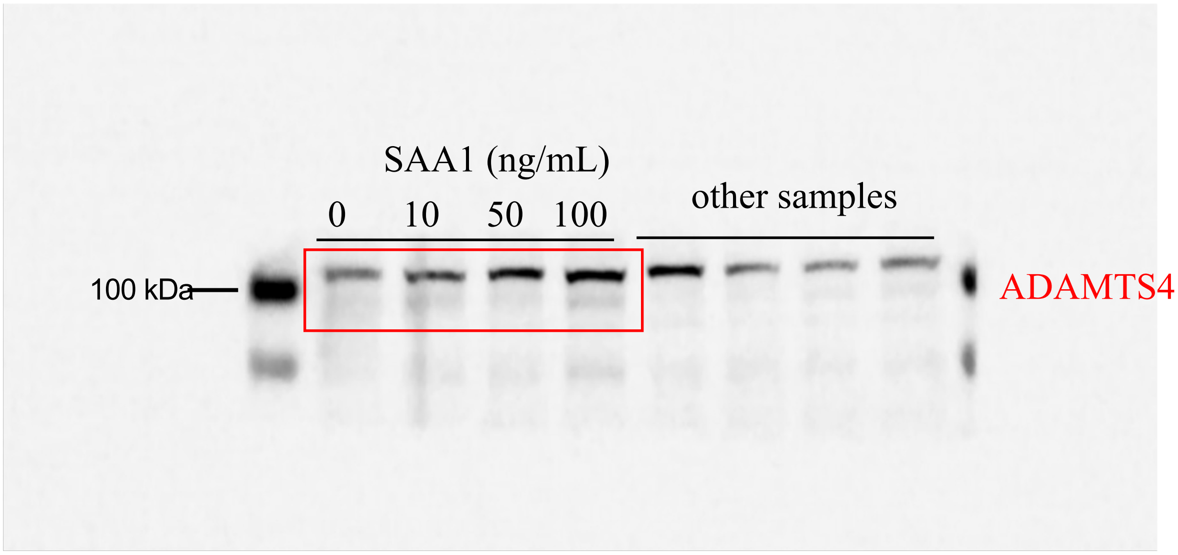

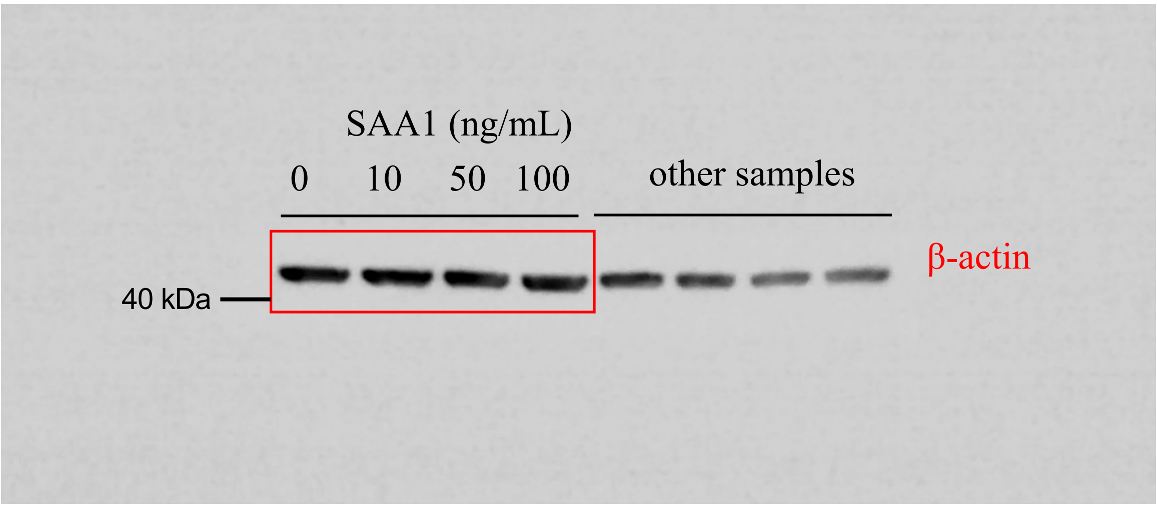

Supplementary Fig. S12: Uncropped and unedited gel image for Fig. 4d.

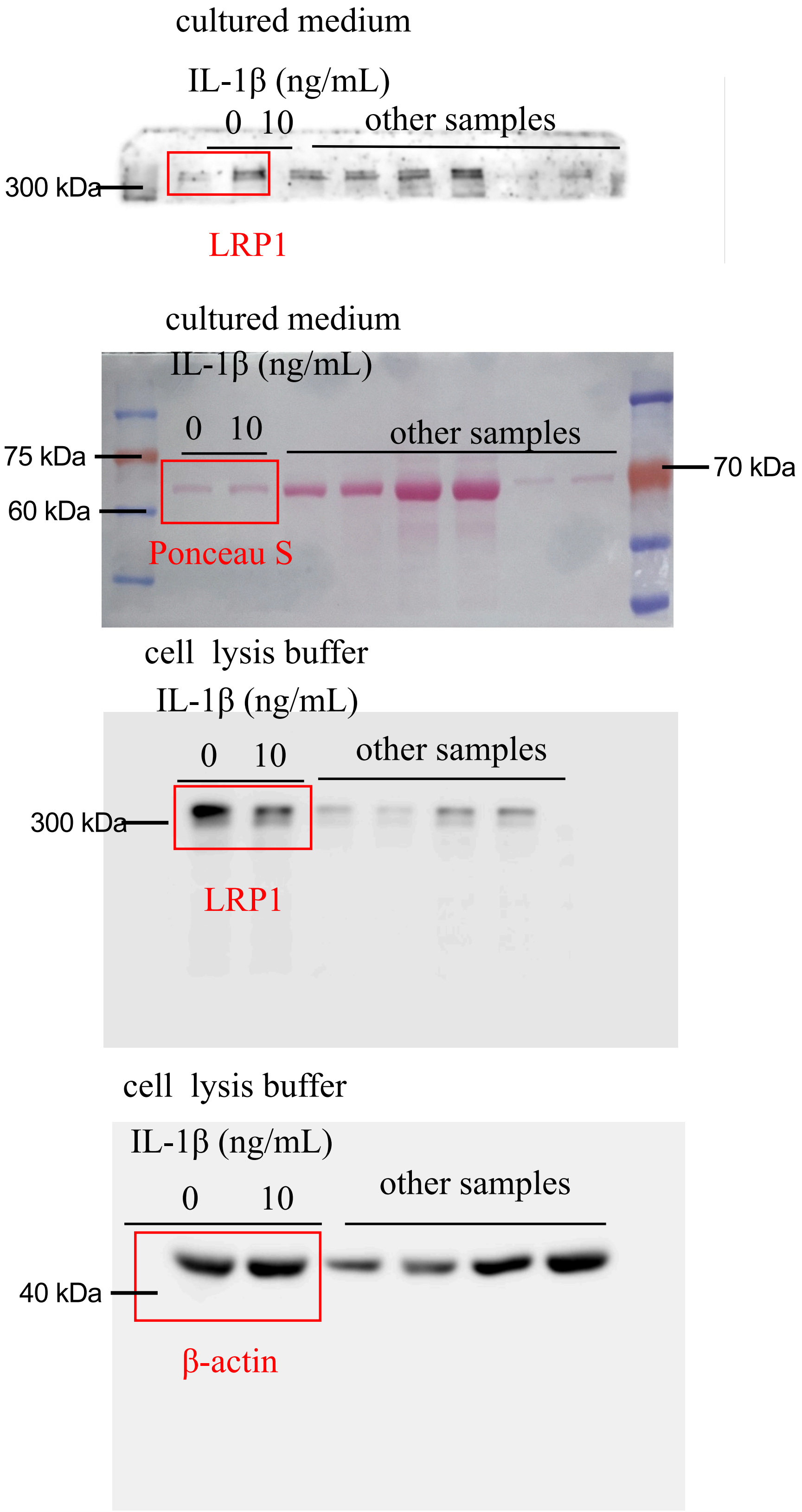

Supplementary Fig. S13: Uncropped and unedited gel image for Fig. 4e.

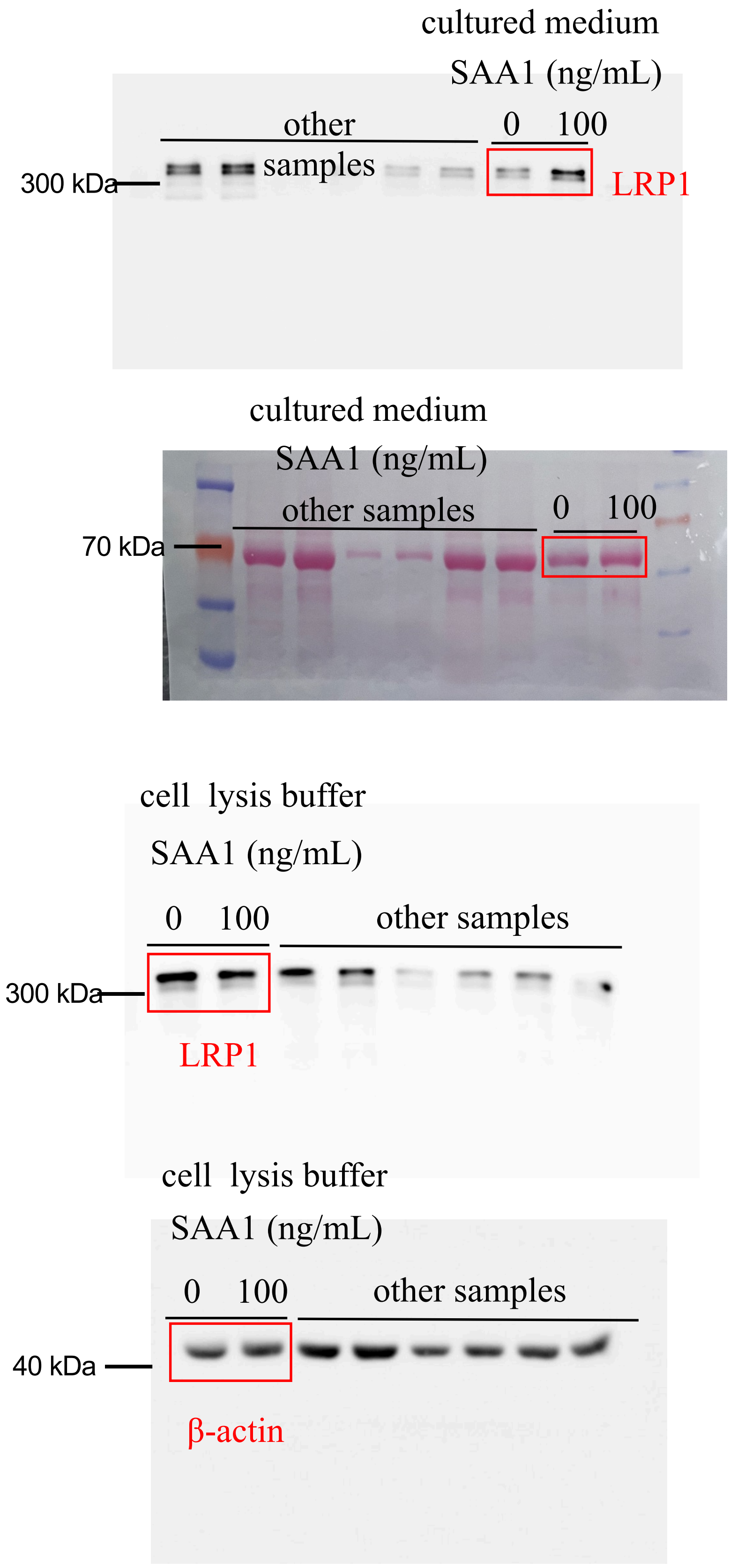

Supplementary Fig. S14: Uncropped and unedited gel image for Fig. 4f.

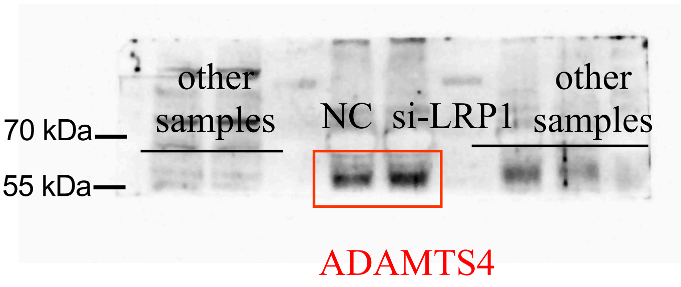

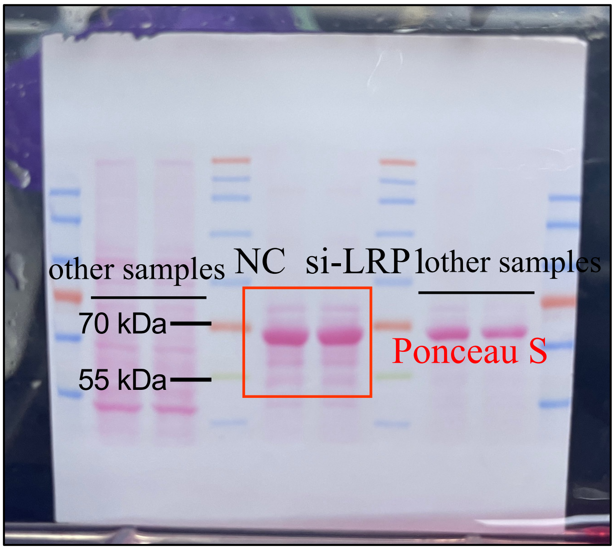

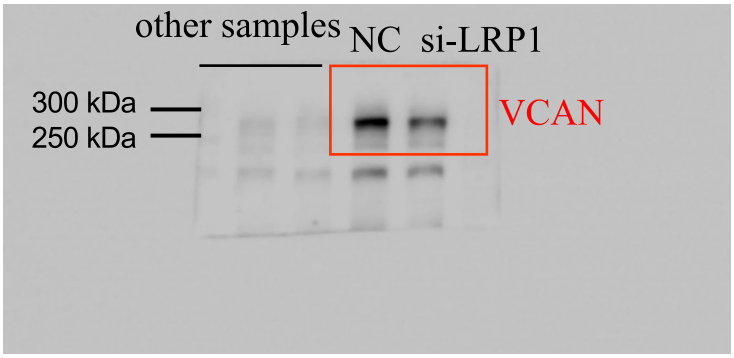

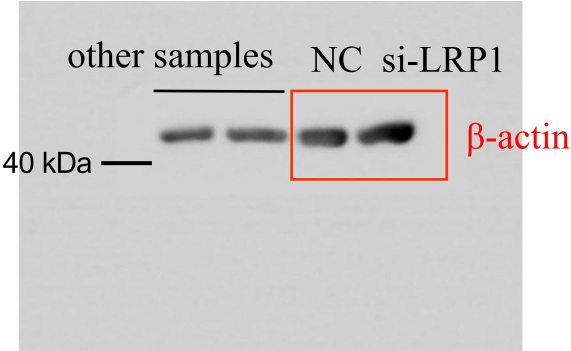

Supplementary Fig. S15: Uncropped and unedited gel image for Fig. 5a.

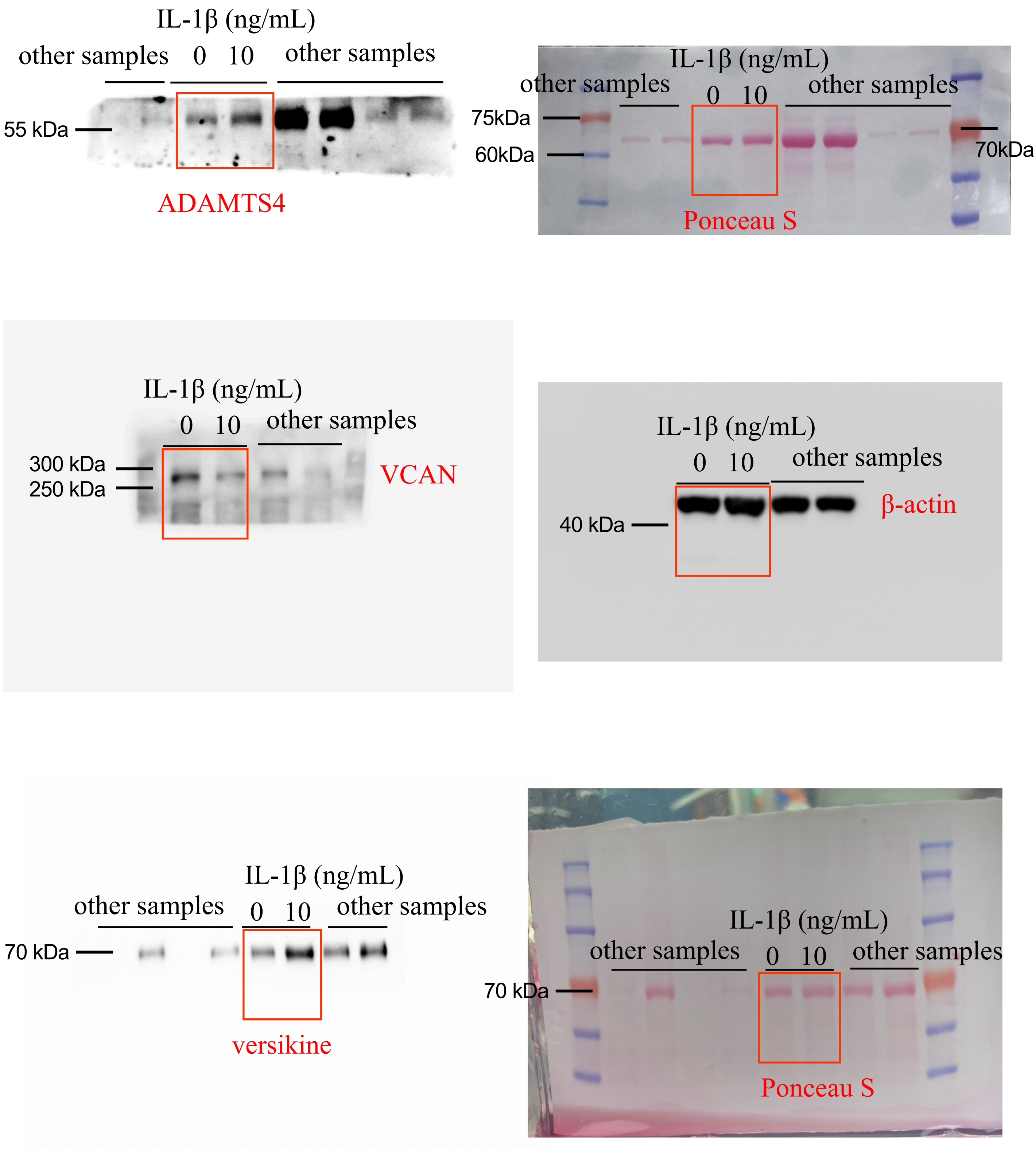

Supplementary Fig. S16: Uncropped and unedited gel image for Fig. 5d.

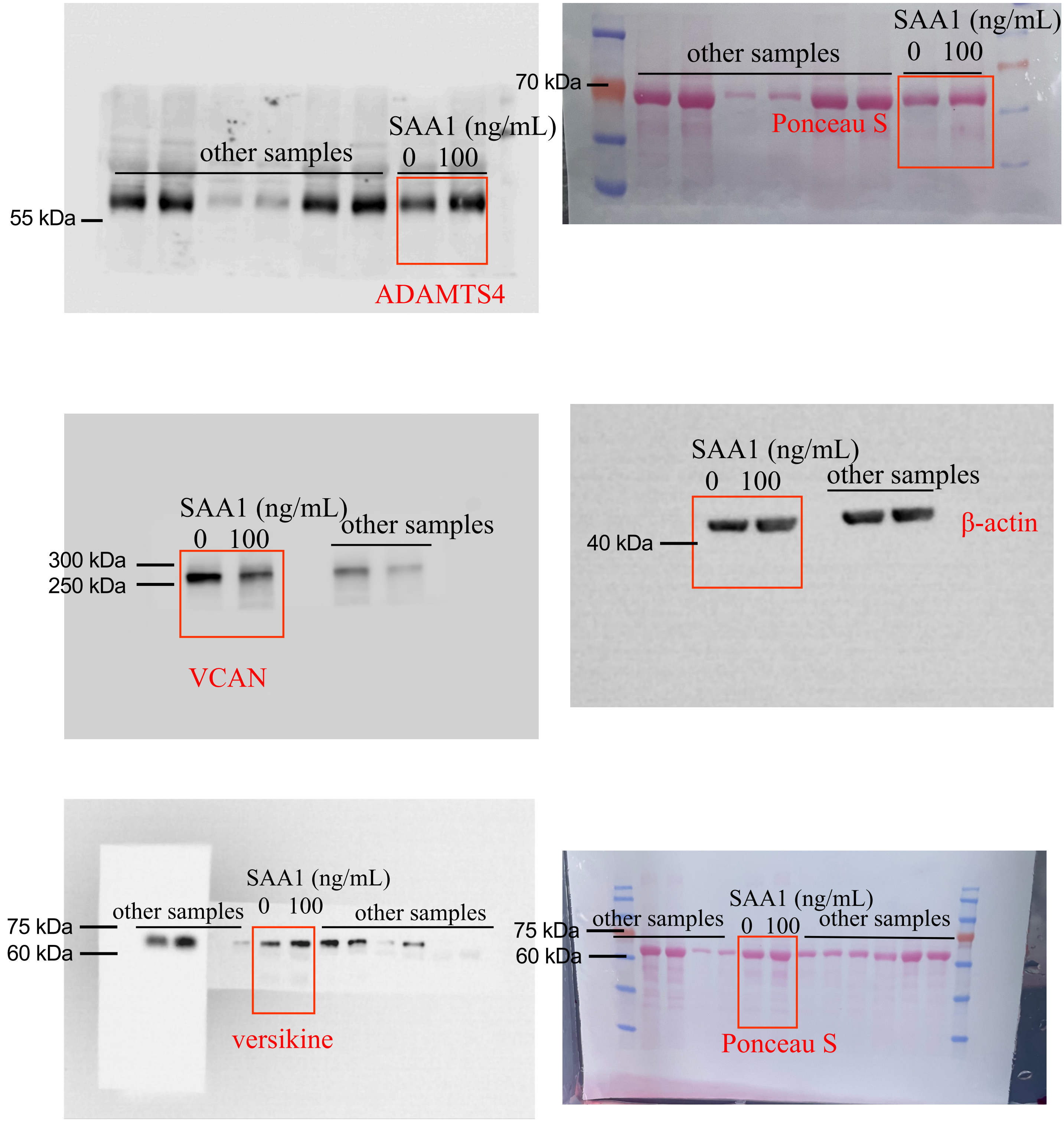

Supplementary Fig. S17: Uncropped and unedited gel image for Fig.5e.

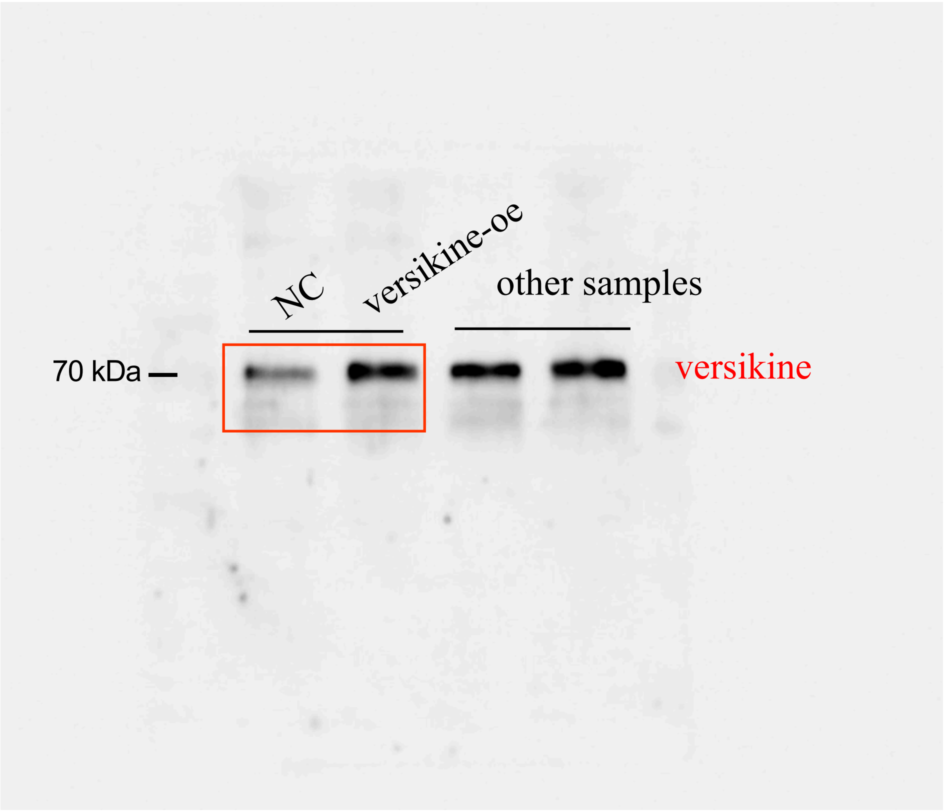

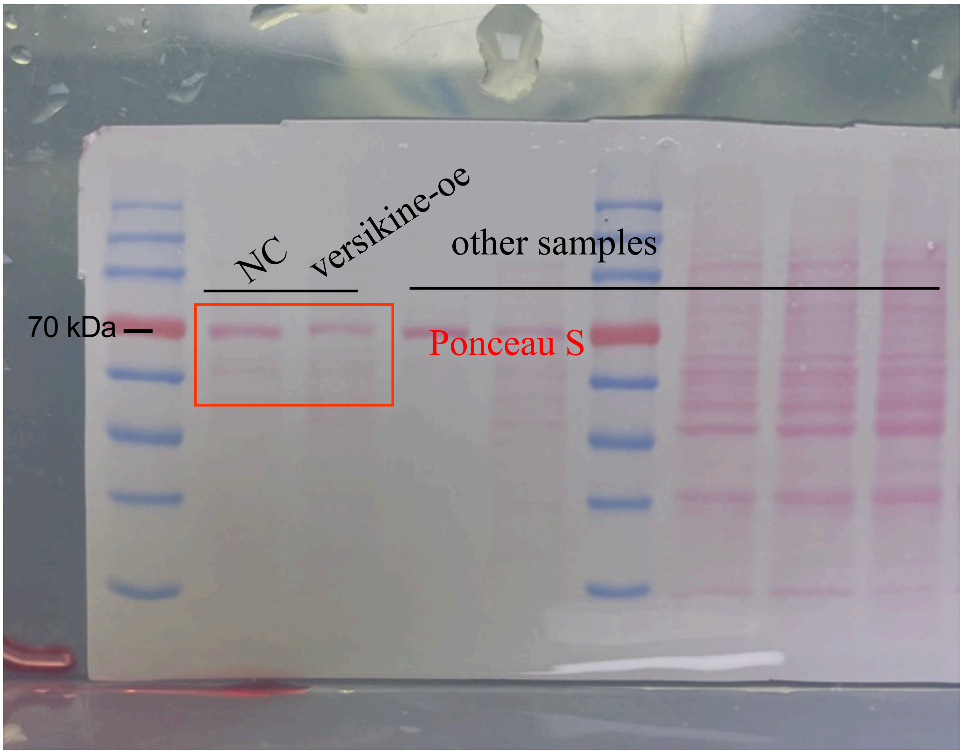

Supplementary Fig. S18: Uncropped and unedited gel image for Fig. 4e.

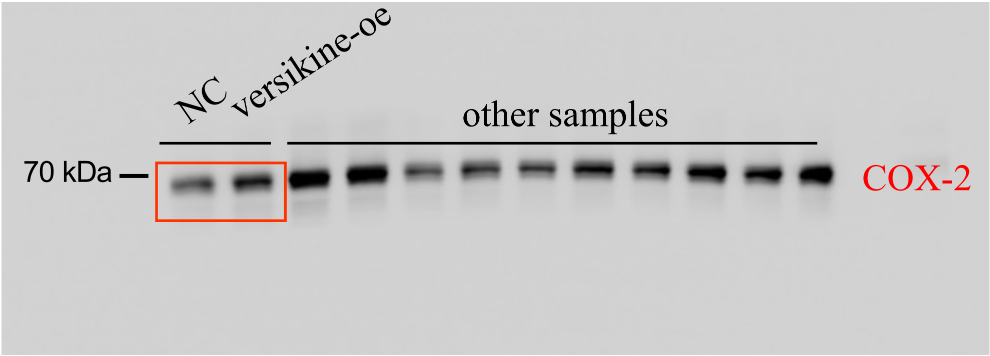

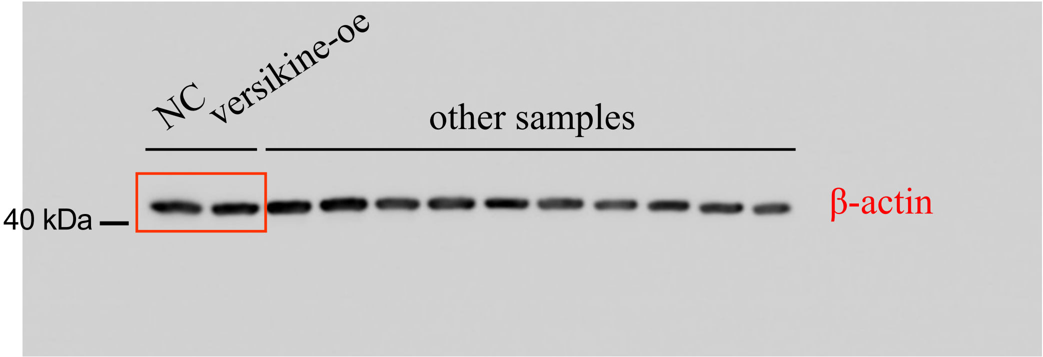

Supplementary Fig. S19: Uncropped and unedited gel image for Fig. 6b.

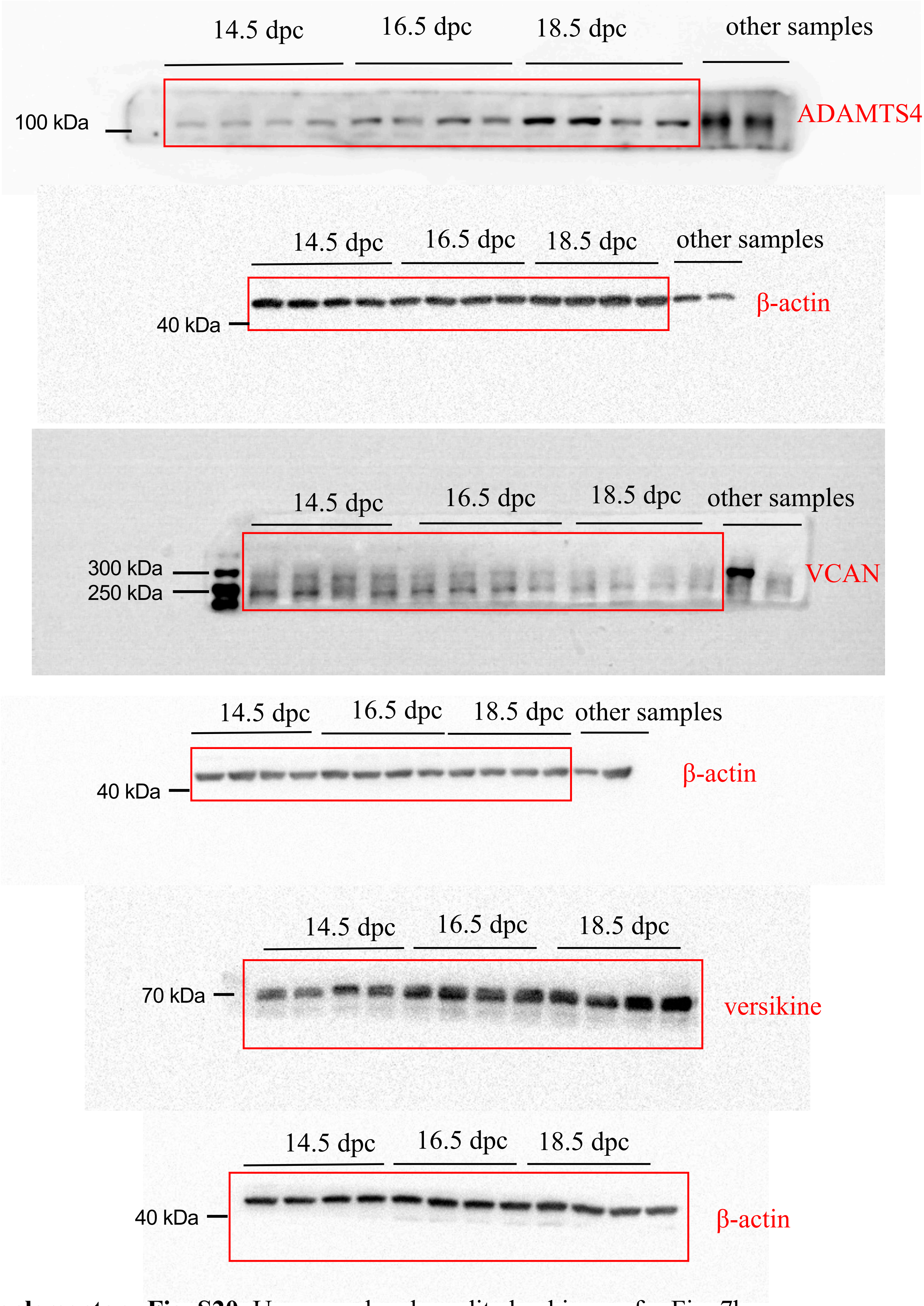

Supplementary Fig. S20: Uncropped and unedited gel image for Fig. 7b.

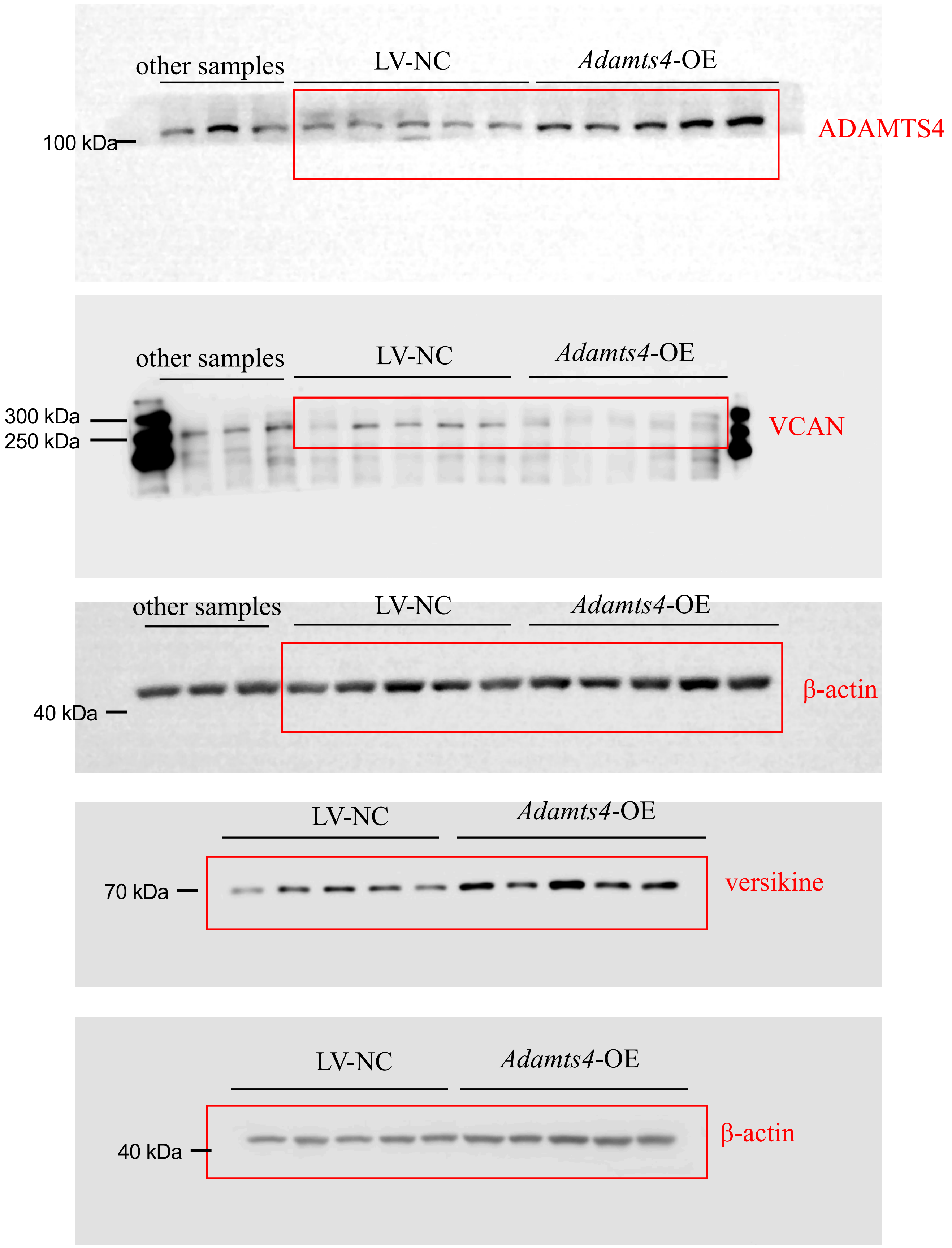

Supplementary Fig. S21: Uncropped and unedited gel image for Fig. 8d.

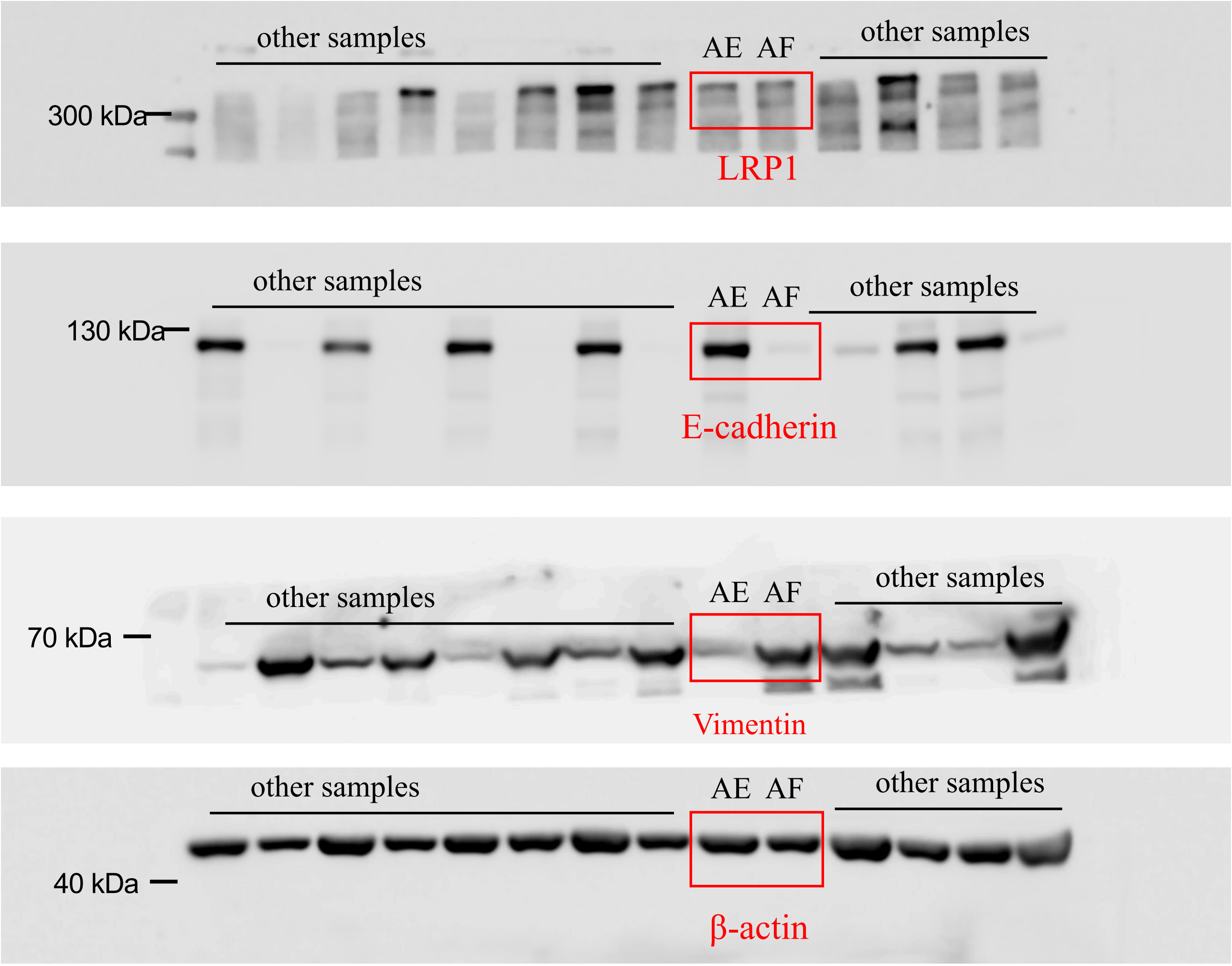

Supplementary Fig. S22: Uncropped and unedited gel image for Fig. S3.

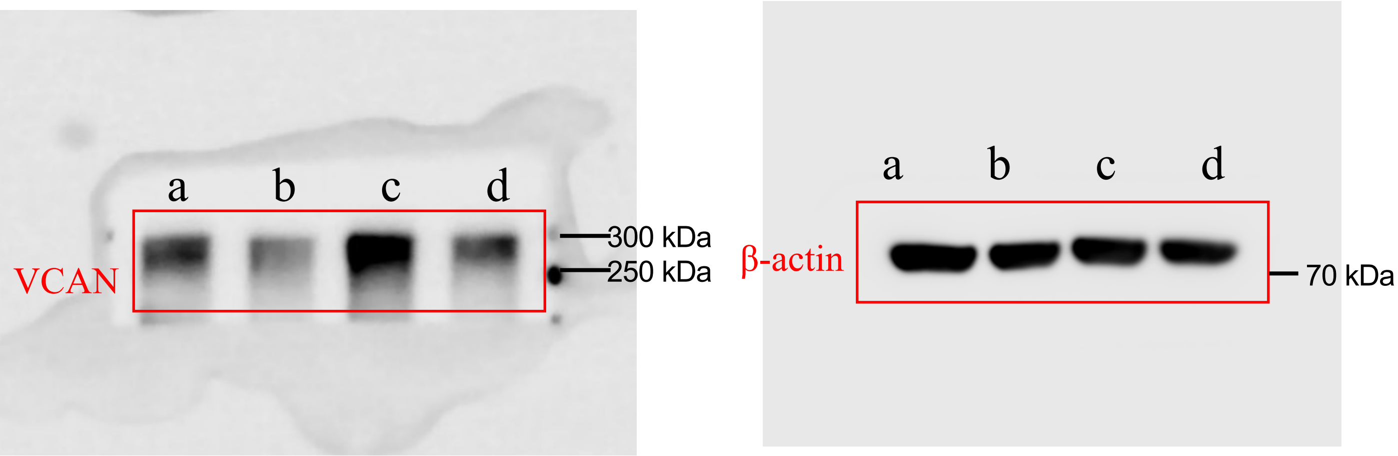

a: control

b: IL-1β (10 ng/mL)

c: ADAMTS4 inhibitor (10 μM)

d: IL-1β+ ADAMTS4 inhibitor

Supplementary Fig. S23: Uncropped and unedited gel image for Fig. S5.

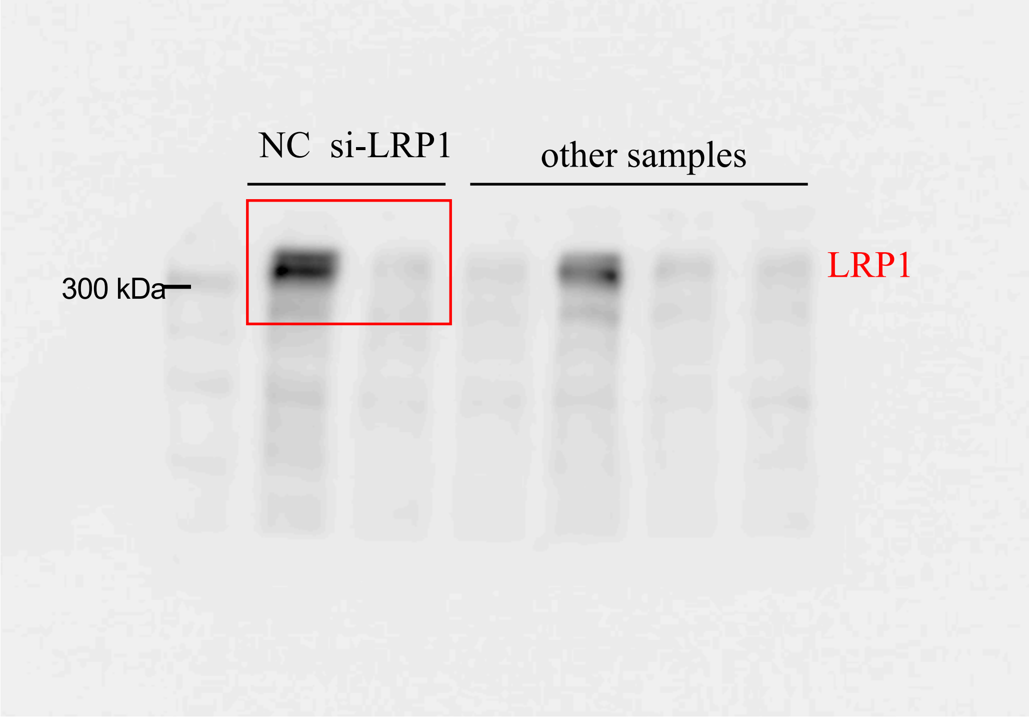

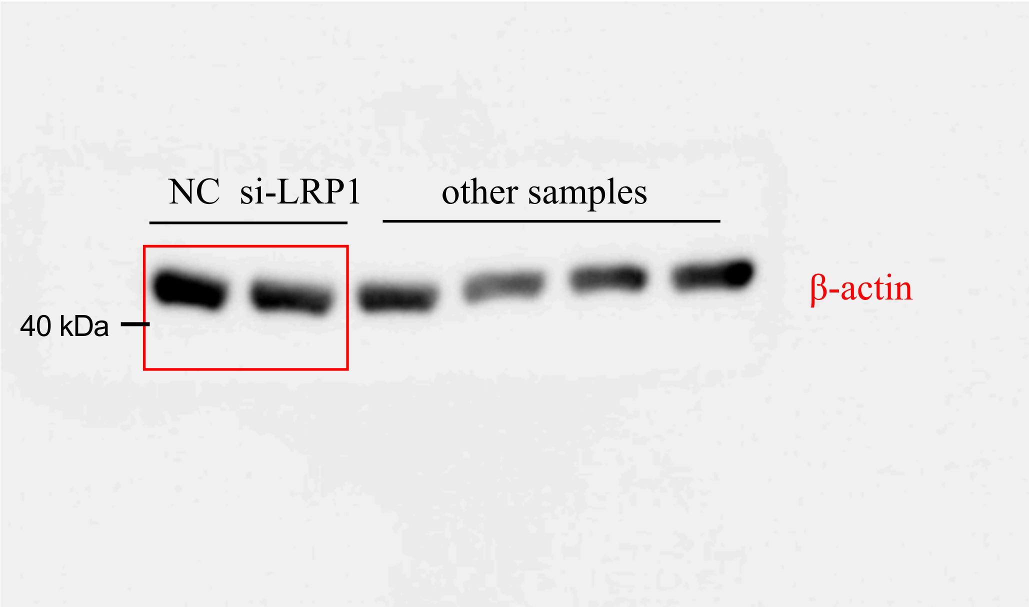

Supplementary Fig. S24: Uncropped and unedited gel image for Fig. S6.

Supplementary Table S1. Primer sequences used in qRT-PCR

Gene	Host	Forward Primer (5'-3')	Reverse primer (5'-3')
VCAN	human	AGTGATGCGGGTCTTTACCG	GCGTCACACTGCTCAAATCC
ACAN	human	ACGCTACACCCTCGACTTTG	ACGTCCTCACACCAGGAAAC
BCAN	human	GCCCCAACGACTCAGGTATC	CAGAGCCCTCTCGGTAGAGAA
NCAN	human	GCCTTGGGCCTTTTGATGC	CCTTGGTCCACTTTATCCGAGG
ADAMTS4	human	CATCCTACGCCGGAAGAGTC	TGCTGCTGCCATCACTGTTA
LRP1	human	ATTGTGTCCCCACACTCGAT	GCCACATATGAAGGAGCCGT
IL1B	human	CCACCTCCAGGGACAGGATA	AACACGCAGGACAGGTACAG
IL6	human	CTTCGGTCCAGTTGCCTTCT	GTGCCTCTTTGCTGCTTTCA
TNF	human	GCTGCACTTTGGAGTGATCG	CTTGTCACTCGGGGTTCGAG
CCL2	human	AGCATGAAAGTCTCTGCCGC	ACACTTGCTGCTGGTGATTCTT
CCL3	human	CGGTGTCATCTTCCTAACCA	GACATATTTCTGGACCCACTC
CCL20	human	TCCTGGCTGCTTTGATGTCA	CAAAGTTGCTTGCTGCTTCTGA
ACTB	human	GCCGACAGGATGCAGAAGGAGATCA	AAGCATTTGCGGTGGACGATGGA
Actb	Mouse	CATTGCTGACAGGATGCAGAAGG	TGCTGGAAGGTGGACAGTGAGG
Il1b	Mouse	TGGACCTTCCAGGATGAGGACA	GTTCATCTCGGAGCCTGTAGTG
Il6	Mouse	TACCACTTCACAAGTCGGAGGC	CTGCAAGTGCATCATCGTTGTTC
Ccl2	Mouse	CAGGTCCCTGTCATGCTTCT	GAGTGGGGCGTTAACTGCAT
Ccl3	Mouse	ACTGCCTGCTGCTTCTCCTACA	ATGACACCTGGCTGGGAGCAAA
Ccl20	Mouse	GTGGGTTTCACAAGACAGATGGC	CCAGTTCTGCTTTGGATCAGCG
Ptgs2	Mouse	GCGACATACTCAAGCAGGAGCA	AGTGGTAACCGCTCAGGTGTTG
