# Supplementary material for: ADAMTS4 is a crucial proteolytic enzyme for versican cleavage in the amnion at parturition
Source: Commun Biol. 2024 Mar 9;7:301. doi: 10.1038/s42003-024-06007-w (PMC10924920; doi:10.1038/s42003-024-06007-w)
Supplement: Supplementary file 5 — Reporting Summary [file 42003_2024_6007_MOESM5_ESM.pdf]

nature portfolio

Corresponding author(s): *Double-anonymous peer review submissions: write DAPR and your manuscript number here instead of author names.*

Last updated by author(s): Oct 12, 2023

Reporting Summary

Nature Portfolio wishes to improve the reproducibility of the work that we publish. This form provides structure for consistency and transparency in reporting. For further information on Nature Portfolio policies, see our Editorial Policies and the Editorial Policy Checklist.

Statistics

For all statistical analyses, confirm that the following items are present in the figure legend, table legend, main text, or Methods section.

n/a	Confirmed	
☐	☒	The exact sample size (*n*) for each experimental group/condition, given as a discrete number and unit of measurement
☐	☒	A statement on whether measurements were taken from distinct samples or whether the same sample was measured repeatedly
☐	☒	The statistical test(s) used AND whether they are one- or two-sided<br>*Only common tests should be described solely by name; describe more complex techniques in the Methods section.*
☒	☐	A description of all covariates tested
☐	☒	A description of any assumptions or corrections, such as tests of normality and adjustment for multiple comparisons
☒	☐	A full description of the statistical parameters including central tendency (e.g. means) or other basic estimates (e.g. regression coefficient) AND variation (e.g. standard deviation) or associated estimates of uncertainty (e.g. confidence intervals)
☐	☒	For null hypothesis testing, the test statistic (e.g. *F*, *t*, *r*) with confidence intervals, effect sizes, degrees of freedom and *P* value noted<br>*Give P values as exact values whenever suitable.*
☒	☐	For Bayesian analysis, information on the choice of priors and Markov chain Monte Carlo settings
☒	☐	For hierarchical and complex designs, identification of the appropriate level for tests and full reporting of outcomes
☒	☐	Estimates of effect sizes (e.g. Cohen's *d*, Pearson's *r*), indicating how they were calculated

Our web collection on statistics for biologists contains articles on many of the points above.

Software and code

Policy information about availability of computer code

Data collection	N/A
Data analysis	N/A

For manuscripts utilizing custom algorithms or software that are central to the research but not yet described in published literature, software must be made available to editors and reviewers. We strongly encourage code deposition in a community repository (e.g. GitHub). See the Nature Portfolio guidelines for submitting code & software for further information.

Data

Policy information about availability of data

All manuscripts must include a data availability statement. This statement should provide the following information, where applicable:

- Accession codes, unique identifiers, or web links for publicly available datasets
- A description of any restrictions on data availability
- For clinical datasets or third party data, please ensure that the statement adheres to our policy

Original data presented in the study can be directed to the corresponding authors.

Research involving human participants, their data, or biological material

Policy information about studies with human participants or human data. See also policy information about sex, gender (identity/presentation), and sexual orientation and race, ethnicity and racism.

Reporting on sex and gender	All participants are female.
Reporting on race, ethnicity, or other socially relevant groupings	All participants are Asians and Han Chinese.
Population characteristics	All patients were normal pregnant women at term (37–40 weeks), aged 25-35 without complications such as gestational diabetes, preeclampsia, and fetal growth restriction.
Recruitment	This study included uncomplicated term (37–40 weeks) pregnancies after elective cesarean section without rupture of fetal membranes and labor or vaginal delivery with spontaneous rupture of fetal membranes.
Ethics oversight	This study was approved by the Ethics Committee of Ren Ji Hospital, School of Medicine, Shanghai Jiao Tong University (approval code: RA-2022-172).

Note that full information on the approval of the study protocol must also be provided in the manuscript.

Field-specific reporting

Please select the one below that is the best fit for your research. If you are not sure, read the appropriate sections before making your selection.

☒ Life sciences ☐ Behavioural & social sciences ☐ Ecological, evolutionary & environmental sciences

For a reference copy of the document with all sections, see nature.com/documents/nr-reporting-summary-flat.pdf

Life sciences study design

All studies must disclose on these points even when the disclosure is negative.

Sample size	No statistical method was used to determine the sample size. Sample size was chosen based on standards in the field. Samples size and number of independent experiments are stated in figure legends or in Methods section.
Data exclusions	No data were excluded from analyses.
Replication	Replication of independent experiments are stated in figure legends or in Statistics and Reproducibility section.
Randomization	Sample/animal allocation were randomized.
Blinding	Blinded when it is possible.

Reporting for specific materials, systems and methods

We require information from authors about some types of materials, experimental systems and methods used in many studies. Here, indicate whether each material, system or method listed is relevant to your study. If you are not sure if a list item applies to your research, read the appropriate section before selecting a response.

Materials & experimental systems

n/a	Involved in the study	
☐	☒	Antibodies
☒	☐	Eukaryotic cell lines
☒	☐	Palaeontology and archaeology
☐	☒	Animals and other organisms
☒	☐	Clinical data
☒	☐	Dual use research of concern
☒	☐	Plants

Methods

n/a	Involved in the study	
☒	☐	ChIP-seq
☒	☐	Flow cytometry
☒	☐	MRI-based neuroimaging

Antibodies

Antibodies used	VCAN (R&D systems; #AF3054), versikine (Thermo Fisher scientific; #PA1-1748A), ADAMTS4 (abcam; #ab185722), ADAMTS4 (Proteintech, #11865-1-AP), LRP1 (ABclonal; #A1439), VCAN (1:1000; ABconal; #A19655) , COX-2 (Cell Signaling Technology, #12282) and β-actin (Proteintech; #60008-1) were used in Western blotting, immunohistochemical or immunofluorescence staining.
Validation	More detailed information about these antibodies ais available on these manufacturers' websites.

Animals and other research organisms

Policy information about studies involving animals; ARRIVE guidelines recommended for reporting animal research, and Sex and Gender in Research

Laboratory animals	C57BL/6 of 10 to 13 weeks
Wild animals	N/A
Reporting on sex	All mice in this study are pregnant female.
Field-collected samples	The study did not involve samples collected from the field.
Ethics oversight	The ethics committee of Ren Ji Hospital, Shanghai Jiao Tong University School of Medicine.

Note that full information on the approval of the study protocol must also be provided in the manuscript.

Plants

Seed stocks	N/A
Novel plant genotypes	N/A
Authentication	N/A
